# Integrated Transcriptomic and Metabolomic Analyses Revealed That Exogenous 24-Epibrassinolide Application Modulated Lignin Biosynthesis in Cold-Stressed *Zizania latifolia*

**DOI:** 10.3390/foods15193392

**Published:** 2026-09-23

**Authors:** Caixia Zhu, Mingxuan Zhang, Zichen Cai, Yukang Gu, Quan Xu, Juan Kan, Man Zhang, Lixia Xiao, Chunlu Qian, Libin Wang

**Affiliations:** 1 Department of Food Science and Engineering, School of Food Science and Engineering, Yangzhou University, Yangzhou 225009, China; zhucaixianh@163.com (C.Z.); kenkirk050301@gmail.com (M.Z.); 15370571156@163.com (Z.C.); 15161393137@163.com (Y.G.); xuquan0103@163.com (Q.X.); kanjuan@yzu.edu.cn (J.K.); mzhang@yzu.edu.cn (M.Z.); lxxiao@yzu.edu.cn (L.X.); 2 College of High Light Industry and Food Engineering, Nanjing Forestry University, Nanjing 210037, China; 3 College of Horticulture, Nanjing Agricultural University, Nanjing 210095, China

**Keywords:** 24-Epibrassinolide, *Zizania latifolia*, lignification, phenylpropanoid biosynthesis

## Abstract

During cold storage, water bamboo (*Zizania latifolia*) is susceptible to lignification. This study employed quality evaluation, transcriptomics, and metabolomics to elucidate the mechanisms by which exogenous 24-epibrassinolide (EBR) delays lignification in water bamboo during cold storage. EBR-treated (1 mg/L) water bamboo was stored at 4 °C for 28 d, followed by additional storage at 25 °C for 3 d. The results indicate that EBR treatment effectively delays postharvest decay in water bamboo while retarding changes in weight loss, hardness, color, and total soluble solids (TSS). Transcriptomic analyses revealed that EBR treatment downregulated the expression of key genes involved in lignin biosynthesis, including peroxidase (POD) and cinnamoyl-CoA reductase (CCR). Simultaneously, EBR suppressed the expression of cell wall degradation-related genes, such as β-glucosidase (β-BGL). Within the lignin biosynthetic diversion pathway, EBR also reduced the expression of UDP-glycosyltransferase 92A1 (UGT92A1), a gene family typically involved in monolignol glycosylation leading to lignin formation. Metabolomic profiling indicated that EBR inhibited the synthesis of metabolites in the phenylalanine biosynthetic precursor pathway, including quinic acid, phenylpyruvic acid, shikimic acid, L-phenylalanine, and (E)-ferulic acid. These findings suggest that EBR prevents lignification and preserves fruit quality in water bamboo by downregulating lignin biosynthesis genes and suppressing phenylpropanoid precursor metabolites.

## 1. Introduction

Water bamboo (*Zizania latifolia*), also known as Chinese wild rice or water bamboo shoots, is a perennial aquatic herbaceous plant of the Poaceae family and constitutes an important aquatic vegetable [1]. It is characterized by high protein content and a rich profile of amino acids, along with essential minerals such as phosphorus, calcium, and iron, as well as vitamins including vitamin C, carotene, thiamine, and riboflavin [2,3], contributing to its widespread consumer acceptance. Cultivation of water bamboo is primarily concentrated in the Yangtze River basin and throughout Southeast Asia. Water bamboo is highly susceptible to cold-induced senescence and deterioration during postharvest cold storage, resulting in flesh hardening, textural degradation, and a significant reduction in commercial value [4].

The active metabolic processes of the cell walls of postharvest bamboo shoots make it difficult for them to maintain their quality during the postharvest stage. This quality deterioration manifests as cell wall thickening and lignin over-accumulation, resulting in lignification [5,6]. Significant lignin accumulation was also reported in fruits and vegetables such as carrots [7], cherries [8], and loquats [9]. Lignin deposition is the primary cause of quality degradation in water bamboo [4]. Low-temperature storage is commonly used to inhibit microbial growth and postharvest physiological processes; however, improper refrigeration temperatures (temperatures below 0–2 °C) trigger stress responses in bamboo plants [10,11]. This condition is mainly manifested as brown discoloration of the succulent stems, pitting, and abnormal activation of the phenylpropanoid metabolic pathway, which leads to lignin deposition and lignification phenomena [12]. This process mainly comes from higher gene expression of lignin synthesis-related enzymes. During lignification, key enzymes in the lignin biosynthetic pathway show a clear increase in both activity and gene expression [13]. This process activates the phenylpropanoid pathway to generate three kinds of lignin monomers, which then promote lignin deposition [14,15]. Meanwhile, reactive oxygen species (ROS) are closely related to plant lignification. H_2_O_2_ levels increase significantly during lignin deposition, so H_2_O_2_ is considered one of the main ROS involved in lignification [16].

Lignin is an abundant aromatic organic polymer in nature, serving as a key structural component of plant cells and fulfilling critical roles in mechanical support and water transport within the cell wall [6,17]. As a highly complex oxygenated phenolic polymer, lignin is formed via radical polymerization of various alcohols, which are oxidized in the cell wall to generate guaiacyl (G), p-hydroxyphenyl (H), and syringyl (S) structural units [18]. The biosynthesis of lignin proceeds primarily via the phenylpropanoid metabolic pathway. This pathway originates from glucose produced by photosynthesis, which is converted into key intermediates of the shikimate pathway, subsequently yielding L-phenylalanine and L-tyrosine as the starting substrates for enzymatic phenylpropanoid metabolism [19,20]. These intermediates subsequently produce L-phenylalanine and L-tyrosine, which are the foundational precursors for the enzymatic biosynthesis of phenylpropanoids. Under the catalytic influence of a series of critical enzymes [21], including phenylalanine ammonia-lyase (PAL), 4-coumaroyl-CoA ligase (4CL), and cinnamyl alcohol dehydrogenase (CAD), plant cells synthesize three monolignol alcohols and transport them to the cell wall. Under the coordinated regulation of H_2_O_2_ [22], peroxidase (POD) and laccase (LAC) are two enzymes that catalyze oxidative polymerization, leading to the formation of lignin polymers [23,24].

Brassinosteroids (BRs) are sterol hormones widely distributed across various plant tissues [25], playing a significant role in promoting plant cell growth, enhancing stress tolerance, and regulating DNA and protein synthesis, while exhibiting unique biological effects on resistance to both biotic and abiotic stresses [26]. 24-Epibrassinolide (EBR) is a brassinosteroid phytohormone. It has been found to stimulate metabolic processes in plants, such as protein and nucleic acid biosynthesis [27]. Furthermore, it improves plant resistance to abiotic stresses, such as salinity, drought, and low temperatures [28,29]. Exogenous EBR treatment significantly increases lignin accumulation in tea plants, thereby conferring enhanced resistance against biotic stress and improving disease resistance [30]. In studies on aquatic vegetables, existing research has shown that EBR treatment clearly inhibits lignin biosynthesis in asparagus [31]. However, current investigations into the preservation of water bamboo have largely centered on exogenous melatonin [32] and nitric oxide [4]. Research on how EBR inhibits browning and preserves the freshness of water bamboo is still limited.

In recent years, integrated transcriptomic and metabolomic analyses have become important tools in vegetable preservation research. These multi-omics approaches provide a full understanding of biological processes, covering everything from gene expression to metabolic changes. Together, they explain the underlying regulatory networks within organisms. Therefore, the present study assessed the effects of exogenous EBR treatment on the postharvest quality of water bamboo during cold storage, thereby evaluating the preservation efficacy of EBR treatment. Furthermore, integrated transcriptomic and metabolomic analyses were employed to elucidate the potential mechanisms by which EBR alleviates postharvest lignification in water bamboo. These findings provide valuable insights into the postharvest regulation of lignification by EBR and offer a theoretical basis for quality preservation in postharvest water bamboo.

## 2. Materials and Methods

### 2.1. Sample Processing

Sample preparation adhered to the methodology previously established by Qian et al. [32], with incidental alterations. Fresh water bamboo (*Zizania latifolia* cv. Xiaolatai) shoots were used as experimental material. Immediately after harvest, shoots were rapidly selected and transported to the laboratory. The uniformly sized and visually identical branches were randomly assigned into two groups (70 branches per group, totaling approximately 11 kg in weight). The branches were immersed in either distilled water (control) or a 1 mg/L EBR solution (treatment) for 30 min, maintaining a plant-to-solution ratio of 1:2 (*w*/*v*). The EBR concentration of 1 mg/L was selected based on preliminary experiments. After the 30 min immersion treatment, water bamboo shoots were air-dried at room temperature for 1 h to remove surface moisture. Shoots from each treatment were then packaged in polyethylene (PE) fresh-keeping bags (dimensions: 30 cm × 20 cm, thickness: 0.03 mm; 5–6 shoots per bag, 750–900 g per pack). The packages were loosely folded to prevent anaerobic fermentation. To simulate commercial retail conditions, shoots after 28 d of cold storage at 4 °C were transferred to 25 °C (RH 86–90%) for an additional 3 d (designated as the 28 + 3 d simulated shelf-life phase). Quality attributes and metabolic changes were comparatively evaluated between CK and EBR groups. Sampling time points were before treatment and on days 7, 14, 21, 28, and 28 + 3 during storage. Epidermal and mesophyll tissue samples of approximately 1 cm in size were excised from fresh sections of each plant; these samples were then snap-frozen in liquid nitrogen and stored at −80 °C. Prior to biochemical assays, the frozen tissue samples were ground into a fine powder in liquid nitrogen using a pre-chilled mortar and pestle. The tissue powder was then immediately homogenized with the appropriate extraction solution for subsequent analyses. Triplicate biological replicates were maintained for each treatment at every sampling time point.

### 2.2. Measurement of Weight Loss Rate

Water bamboo samples were weighed using an electronic balance (ZG-TP203, ZOGGI Songjing, Yongkang, China) and calculated using the formula:

Weight loss rate (%) = (Pre-storage water bamboo weight − Post-storage water bamboo weight)/Pre-storage water bamboo weight × 100%

### 2.3. Total Soluble Solids Content and Firmness Determination

Total soluble solids (TSS) content of the water bamboo samples was measured using a hand-held refractometer (DFT-F10V55H23, Shenzhen Digitizing Fluid Technology Co., Ltd., Shenzhen, China) and expressed as a percentage by mass.

Epidermal firmness at the middle section of the water bamboo was determined using a GY-1 type texture analyzer (Zhejiang TOP Instrument Co., Ltd., Hangzhou, China) equipped with a cylindrical probe (diameter 0.4 cm). The probe was inserted vertically to a depth of 0.4 cm, and firmness was recorded in units of kg·cm^−2^.

### 2.4. Color Difference Value Measurement

Using a handheld colorimeter (LS172, Shenzhen Linshang Technology Co., Ltd., Shenzhen, China), L* (lightness), a* (red-green value), and b* (yellow-blue value) of the middle peel region of water bamboo were measured, with a standard white board used as a reference. To figure out the total color difference (Δ*E*), we used the formula below, where L_0_*, a_0_*, and b_0_* represent the reference values.

(1) $$\Delta E=\sqrt{{\left(L^{*}-{L_{0}}^{*}\right)}^{2}+{\left(a^{*}-{a_{0}}^{*}\right)}^{2}+{\left(b^{*}-{b_{0}}^{*}\right)}^{2}}$$

### 2.5. Lignin Staining

The samples were stained using the phloroglucinol-hydrochloric acid method [33]. Transverse sections were prepared from the basal tissue of both groups. A 500 μL aliquot of 1% phloroglucinol solution was uniformly applied to each cross-section, and after 5 min of staining, concentrated HCl was added to complete the Wiesner reaction. Lignin deposits exist in the form of reddish-brown spots.

### 2.6. In Situ Detection of Superoxide Anion and Hydrogen Peroxide

The staining method was slightly modified from Liu et al. [34]. Transverse sections approximately 0.6–1 cm thick were taken from the basal tissue of water bamboo and incubated in the dark for 1–2 h in 0.5 mg/mL nitroblue tetrazolium (NBT) solution (dissolved in 50 mM phosphate buffer, pH 7.8) and a 1 mg/mL 3,3′-diaminobenzidine (DAB) solution (prepared in 50 mM citrate-phosphate buffer, pH 3.8). The stained sections were then rinsed with 70% ethanol and sterile distilled water. Superoxide anion (O_2_^−^) appeared as blue formazan deposits at NBT precipitation sites, while H_2_O_2_ produced reddish-brown or dark brown polymer precipitates at DAB polymerization sites. The study used ImageJ (version 1.52p, National Institutes of Health, Bethesda, MD, USA) to measure color intensity.

### 2.7. RNA-Seq Analysis

Randomly selected water bamboo shoots were collected at 0, 14, and 28 d of storage for transcriptome analysis. Fresh water bamboo tissue was flash-frozen in liquid nitrogen and stored at −80 °C to preclude secondary alterations in gene expression. RNA extraction was performed following Qian et al. [33]. RNA integrity assessment, library construction, and sequencing were conducted according to the BMKCloud platform protocols. The research was conducted on the Illumina NovaSeq platform for sequencing. Clean reads were aligned to the water bamboo genome (https://ngdc.cncb.ac.cn/gwh/Assembly/22880/show, accessed on 20 April 2026) using HISAT2 software(version 2.0.4). Gene expression levels were quantified using the FPKM metric, which accounts for gene length and the number of reads per million mapped reads aligned to exon models. Differential expression analysis was performed with DESeq2 and edgeR, applying a threshold of |log2(Fold Change)| > 1 and False Discovery Rate (FDR) < 0.01 to identify differentially expressed genes (DEGs). Pearson correlation coefficient (*r*) and principal component analysis (PCA) were used to assess sample correlation and reproducibility, with *r* values closer to 1 indicating stronger correlation between replicates. For each designated comparison group, results including gene expression levels, lists of DEGs, and functional enrichment analyses were obtained. FPKM values were visualized as heatmaps using the heatmap package in R software(version 3.6.1). The study collected three biological replicate samples (n = 3) for RNA-seq analysis.

### 2.8. Metabolomics Analysis

Metabolomic analysis was conducted in collaboration with the BMKCloud platform, using an exploratory broad-target metabolomics approach on the same samples as those subjected to transcriptomic analysis, enabling qualitative and quantitative profiling of metabolites. The analytical platform comprises a Waters Acquity I-Class PLUS UHPLC system interfaced with an AB Sciex QTRAP 6500+ high-sensitivity mass spectrometer (SCIEX, Concord, Ontario, Canada). The chromatographic column uses Waters HSS-T3 (1.8 µm, 2.1 mm * 100 mm). Qualitative and quantitative mass spectrometric analysis of metabolites was performed using the in-house database GB-PLANT. Following metabolite identification and quantification, data quality assessment, annotation analysis, differential expression analysis, and functional enrichment were carried out. The default criteria for differential metabolite screening were set at Variable Importance in Projection (VIP) > 1 and *p* < 0.05. PCA was used to judge the repeatability of the samples within the group and the quality control samples. The identified compounds were searched for classification and pathway information in KEGG databases. Three biological replicates (*n* = 3) were separately collected for subsequent metabolomics analysis.

### 2.9. Data Analysis

Statistical analyses were performed using SPSS 27.0 (IBM, Armonk, NY, USA). All experiments were conducted in triplicate, and data are presented as the mean ± standard deviation (SD). The experimental design included two sources of variation: treatment (CK vs. EBR) and storage time (0, 7, 14, 21, 28, and 28 + 3 d). A two-way analysis of variance (Two-way ANOVA) was performed to evaluate the main effects of treatment, storage time, and their interaction (Treatment × Storage time). Differences among group means were analyzed using one-way ANOVA followed by Tukey’s honestly significant difference (HSD) test at a significance level of *p* < 0.05. All experiments were carried out with a completely randomized design. All experimental assays were conducted using at least three independent biological replicates (*n* = 3). Relationship visualization was conducted using Origin 2024.

## 3. Results

### 3.1. Appearance and Quality Changes Caused by EBR Treatment

Postharvest cold storage observations of water bamboo revealed distinct phenotypic changes over the storage period (Figure 1A). Both treatment and CK groups exhibited quality deterioration, including browning and shriveling. Compared with the CK group, the EBR-treated samples displayed generally lower degrees of shriveling and senescence-associated browning, along with superior external appearance. This phenotypic difference became more pronounced after 14 days of storage. However, by the 28 + 3 d time point, the delaying effect was no longer pronounced.

A significant correlation was detected between the rate of weight loss and storage duration in water bamboo (Figure 1B-a). Over the storage period, weight loss rates in the EBR-treated group were consistently and significantly lower than those in the CK group, reaching maximum values of 39.0% and 36.5% at 28 + 3 d for CK and EBR, respectively. Firmness and TSS declined progressively with storage duration (Figure 1B-b,c). From 14 d onward, the EBR group exhibited superior retention of firmness and TSS; across the entire storage period, both parameters were significantly enhanced by EBR treatment. The Δ*E* value increased gradually over time. From the mid- to late-storage phase (7 d to 28 + 3 d), Δ*E* rose to 26.8 in the CK group, whereas EBR treatment suppressed it to 25.5, effectively delaying surface discoloration (Figure 1B-d). From day 21 to day 28 + 3, EBR treatment significantly suppressed weight loss (Figure 1B-a), retarded firmness increase (Figure 1B-b), and maintained higher TSS levels (Figure 1B-c) compared with the control.

### 3.2. Analysis of Staining Results

Figure 2 depicts the deposition of cell wall lignin, O_2_^−^, and H_2_O_2_ in water bamboo during storage. Following EBR treatment, the number of brown spots in water bamboo slices was lower than in the CK group, with the most pronounced difference observed at 28 d. The levels of O_2_^−^ and H_2_O_2_ generally increased progressively with prolonged storage time (Figure 1A and Figure 2B,C). Compared with the CK group, EBR treatment resulted in markedly reduced color deposition, suggesting that EBR may inhibit excessive accumulation of ROS. Semi-quantitative image analysis further confirmed that EBR treatment significantly reduced the staining intensity of O_2_^−^ by 38.5% at 28 d and H_2_O_2_ by 41.8% at 28 + 3 d compared with the control, consistent with the visual suppression of oxidative browning.

### 3.3. Transcriptome Analysis

#### 3.3.1. Gene Expression Analysis

To examine the effects of EBR treatment on gene expression in water bamboo, samples from both groups at 0, 14, and 28 d were subjected to RNA-sequencing (RNA-seq) analysis. A total of 95.49 Gb of clean data were obtained from RNA sequencing, with each sample yielding 6.12 Gb of clean data, and the Q30 base percentage consistently exceeding 95.17%, confirming the high quality of the sequencing data for subsequent analyses. Alignment of the Clean Reads from each sample against the water bamboo reference genome yielded mapping efficiencies ranging from 76.27% to 90.24%. As shown in Figure 3A, the pairwise correlation coefficients among samples are markedly high, with the vast majority exceeding 0.7, indicating substantial similarity between different samples. Figure 3B presents the PCA, which reveals the major differences among samples following dimensionality reduction. The five groups display clear separation, suggesting that the distinct treatments resulted in significant divergence among the groups.

#### 3.3.2. Identification and Enrichment of DEGs

The study compared DEGs between different groups. The number of DEGs varied clearly across groups. The highest number of DEGs was observed between the CK0 and EBR14 groups, indicating that EBR treatment induced a pronounced response at the early stage of storage. The fewest DEGs were identified between the CK14 and EBR14 groups, suggesting that gene expression profiles in the two groups were similar at the middle stage of storage (Figure 4C). Only one co-expressed DEG was found across all comparison groups, implying distinct regulatory characteristics under different treatment conditions (Figure 4D). This study focused on key genes involved in lignin biosynthesis, with particular emphasis on the comparison between the CK28 and EBR28 groups. A total of 608 DEGs were identified, comprising 212 upregulated and 396 downregulated genes. The volcano plot (Figure 4A) and cluster analysis (Figure 4B) further illustrated the expression patterns of these DEGs, revealing pronounced differences in gene expression between the two groups, with clear directionality of upregulation and downregulation at the transcriptional level.

For the CK28 and EBR28 groups, the number of annotated DEGs was approximately 553, of which 420 DEGs were annotated by Gene Ontology (GO) analysis and 382 by Kyoto Encyclopedia of Genes and Genomes (KEGG) analysis. The GO analysis grouped them into three main categories. DEGs were predominantly enriched in molecular functions and biological processes, with the majority of DEGs being significantly downregulated (Figure 5A). The principal molecular function categories included cellular processes and metabolic processes; cellular components involved cellular anatomical entities; and biological processes encompassed transport activities and catalytic activities. KEGG analysis indicates that the enriched pathways of the DEGs are predominantly in glycolysis/gluconeogenesis (ko00010), carbon fixation in photosynthetic organisms (ko00710), carbon metabolism (ko01200), selenocompound metabolism (ko00450), and phenylpropanoid biosynthesis (ko00940) (Figure 5B).

### 3.4. Metabolomic Analysis

#### 3.4.1. Metabolite Quantification

Metabolomics detected a total of 2353 metabolites. Among them are 375 lipids, 299 ketones, aldehydes, and esters, 234 terpenoids, 211 carbohydrates, 202 amino acids, 174 organic acids, 119 flavonoids, 147 alkaloids, 76 polyphenols, 76 alcohols, 67 nucleotides, 38 coumarins, 37 phenylpropanoids, 19 quinones, 18 lignans, 12 vitamins, 30 steroids and their derivatives, and 219 other metabolites (Figure 6A). PCA was performed on the five sample groups; PC1 and PC2 accounted for 39.31% and 18.66% of the variance among samples, respectively, with the two principal components cumulatively explaining nearly 58% of the total data variation (Figure 6B).

#### 3.4.2. KEGG Pathway Analysis

Based on the CK28 and EBR28 groups, metabolomic profiling identified 456 differential metabolites (DEMs), among which 235 were upregulated and 221 were downregulated. The DEMs were significantly enriched in pathways related to phenylalanine, tyrosine, and tryptophan biosynthesis, phenylpropanoid metabolism, and phenylalanine biosynthesis (Figure 7). The DEMs enriched within these pathways may influence lignin production and secondary metabolite synthesis during cold storage of water bamboo. Notably, DEMs enriched in the phenylalanine biosynthesis pathway may directly participate in regulating lignin synthesis.

### 3.5. DEGs and DEMs Associated with the Phenylalanine Biosynthetic Pathway

Eight related metabolites and twelve key regulatory genes implicated in the lignin biosynthetic pathway were identified through integrated transcriptomic and metabolomic KEGG pathway enrichment analyses (Figure 8, Table 1). In the downstream steps of lignin biosynthesis, the expression levels of cinnamoyl-CoA reductase (CCR) and β-glucosidase (β-BGL) were downregulated, while POD displayed inconsistent upregulation and downregulation. Furthermore, in the lignin monomer biosynthetic branch, UDP-glycosyltransferase 92A1 (*UGT92A1*) expression was markedly reduced after EBR treatment. Together, these findings suggest that lignin biosynthesis may be inhibited.

EBR treatment also significantly reduced the levels of multiple aromatic amino acids and their precursors, including D-(-)-quinic acid, L-tyrosine, phenylpyruvic acid, protocatechuic acid, shikimic acid, L-phenylalanine, L-tryptophan, and (E)-ferulic acid as well as its derivatives. D-(-)-quinic acid and shikimic acid are intermediates of the shikimate pathway; D-(-)-quinic acid can be converted to shikimic acid, thereby providing precursors for subsequent aromatic amino acid synthesis and contributing to the production of phenolic or antioxidant compounds. L-tyrosine, L-phenylalanine, and L-tryptophan are aromatic amino acids; phenylpyruvic acid is the transamination product of phenylalanine, and protocatechuic acid represents a branch of phenylpropanoid metabolism. L-phenylalanine serves as a precursor for phenylpropanoid metabolism, while (E)-ferulic acid directly participates in lignin biosynthesis. The marked reductions in the levels of these compounds are a direct consequence of EBR-mediated suppression of lignin synthesis. Notably, L-phenylalanine is synthesized via the shikimate pathway, and a concurrent decline in shikimic acid and D-(-)-quinic acid levels was observed. Collectively, these results preliminarily suggest that EBR primarily inhibits lignin accumulation in water bamboo by suppressing or reducing the generation of phenylpropanoid precursors and the expression of genes specific to the lignin biosynthetic pathway.

## 4. Discussion

The present study systematically investigates the inhibitory effects of EBR treatment on quality deterioration in water bamboo during cold storage. Studies have shown that the respiration rate of postharvest bamboo shoots is extremely high, which in turn affects the changes in related quality indicators and also provides energy and signals for the lignification process [35,36]. EBR treatment effectively delays browning, softening, and weight loss through multiple regulatory mechanisms, while also maintaining TSS content (Figure 1). During cold storage, water bamboo gradually undergoes browning and softening accompanied by wilting, a process closely associated with weight loss, changes in firmness, and alterations in surface color [37,38,39]. In this study, compared with the CK group, water bamboo treated with EBR remained fresh after more than 20 d of storage, with no significant color change, shrinkage, or water loss, indicating that EBR treatment can effectively delay browning and lignification, thereby enhancing fruit firmness and plumpness. Previous research has demonstrated that EBR mitigates fruit browning and reduces water loss by enhancing cell membrane integrity, consequently reducing weight loss [40]. The effects of EBR treatment in reducing losses of firmness and TSS content have also been reported in grapes [41]. The present study corroborates these findings with respect to EBR-mediated delay of quality deterioration in water bamboo. Similar effects of EBR treatment have been confirmed in zucchini [42]. During postharvest storage of water bamboo, ROS accumulate within fruit cells, inducing oxidative stress and accelerating lignification and senescence [28]. In this study, EBR treatment activated the antioxidant system, resulting in reduced accumulation of H_2_O_2_ and O_2_^−^, which were negatively correlated with those observed in the CK group (Figure 2). This suggests that EBR may counteract ROS-induced oxidative damage and reduce cellular membrane injury. This finding has been corroborated in studies on kiwifruit [43] and lotus root [44].

Under normal physiological conditions, the phenylpropanoid metabolic pathway provides plants with structural support and substances that defend against external damage. However, under postharvest stress, this pathway is aberrantly activated, leading to lignification in fruits and vegetables [45]. In this study, four genes associated with lignin biosynthesis were identified: POD, CCR, β-BGL, and *UGT92A1*. EBR treatment suppressed the gene expression of key downstream enzymes in the phenylpropanoid pathway at the transcriptional level, and these genes may serve as critical candidate targets for inhibiting postharvest lignification, thereby providing a plausible explanation for the aforementioned staining and phenotypic observations. A previous study reported that EBR application downregulated genes related to cell wall synthesis and upregulated phenylpropanoid metabolism-related genes, thereby attenuating softening during postharvest storage of blueberries [46]. Previous studies have demonstrated that asparagus senescence is linked to the phenylpropanoid pathway, and EBR suppresses asparagus lignification by downregulating the expression of PAL, C4H, 4CL, COMT, CCR, CAD, and POD [31]. Notably, whereas EBR typically activates defensive secondary metabolism, such as promoting lignin synthesis, under stress conditions, in the present study, EBR exhibited a specific inhibitory effect on the lignin biosynthesis pathway [30].

Lignin biosynthesis is a complex enzymatic process [47]. CCR is the pivotal enzyme responsible for reducing cinnamoyl-CoA to cinnamyl alcohol, with its activity exerting a decisive influence on the ratio and total content of lignin monomers [48]. The present study reveals that the CCR gene is significantly downregulated under EBR treatment, indicating that EBR directly interferes with the rate-limiting step of lignin monomer biosynthesis. β-BGL hydrolyzes monolignol glycosides, such as coniferyl alcohol glucoside, during lignin synthesis, thereby releasing lignin monomers. Inhibition of β-BGL may compromise the conversion of soluble sugars in the cell wall into lignin precursors [49]. *UGT92A1* belongs to the UDP-glycosyltransferase family and typically participates in the glycosylation of monolignols or the deactivation of hormones [50]. The study found that *UGT92A1* expression was downregulated, suggesting that this alteration may redirect the flux of the phenylpropanoid metabolic pathway toward alternative non-lignin routes. UGT family members are crucial for regulating how plants respond to stress and how they build up secondary metabolites [51]. In citrus fruits, it has been reported that the UGT family is involved in the lignin synthesis pathway, and its expression is positively correlated with lignin synthesis [52]. POD catalyzes the oxidative polymerization of monolignols (such as coniferyl alcohol and p-coumaryl alcohol) to form a high-molecular-weight lignin network [53]. The downregulation of POD was less pronounced. Given the catalytic role of POD in the oxidative polymerization of lignin monomers, its reduced expression indicates that EBR has an inhibitory effect on the lignin synthesis pathway. Corroborating evidence from studies on loquat [9] and pear [53] demonstrates that inhibiting either POD or CCR activity effectively delays lignification during fruit and vegetable preservation. In other plants, EBR can coordinately regulate the expression of multiple phenylpropanoid pathway enzymes, such as PAL and 4CL, thereby exerting precise control over lignin biosynthesis [54].

Metabolomic analysis revealed a more upstream mechanism. EBR treatment significantly decreased the levels of various aromatic amino acids and their precursors, which is consistent with findings in asparagus [31]. L-phenylalanine, a direct precursor metabolite in the phenylpropanoid metabolic pathway, and (E)-ferulic acid, an intermediate in lignin biosynthesis, showed significant reductions in content, directly resulting from EBR’s inhibition of lignin biosynthesis. L-phenylalanine is generated by the shikimate pathway. The simultaneous decrease in shikimic acid and D-(-)-quinic acid levels within the shikimate pathway observed in this study suggests that EBR may limit the shikimate pathway upstream, aligning with the shikimate pathway’s role in synthesizing aromatic amino acids, which in turn activate the phenylpropanoid pathway [55,56]. The reduction in intermediates such as phenylpyruvic acid and protocatechuic acid further corroborates the blockage of phenylalanine conversion towards lignin. Combined transcriptomic and metabolomic analyses indicated that both involved the phenylalanine biosynthesis pathway, with the downregulation of metabolites also pertaining to the upstream shikimate pathway, corresponding to the downregulation of key enzyme genes in the transcriptome (POD, CCR, β-BGL, *UGT92A1*).

Overall, the multi-omics and quality indicator evidence in this study constructed a comprehensive regulatory network. Through this network, EBR can alleviate the lignification of water bamboo during postharvest cold storage, thereby achieving a preservation effect. These findings suggest that postharvest EBR immersion treatment is a promising and practical postharvest technology that can extend the marketability of water bamboo and reduce economic losses in this industry. From an applied postharvest perspective, while the laboratory-scale protocol was optimal for elucidating these regulatory mechanisms, several limitations regarding industrial scalability must be considered. On a commercial scale, prolonged immersion treatments entail high operational bottlenecks and chemical waste. Consequently, advancing from dipping to spray-based applications, such as electrostatic or automated atomization systems, represents a more practical and feasible methodology for large-scale industrial adoption. Future studies should focus on optimizing spray parameters and evaluating hormone residue dynamics to facilitate the commercial translation of EBR-based preservation technologies.

## 5. Conclusions

In conclusion, exogenous EBR (1 mg/L) treatment effectively maintained the postharvest commercial quality of cold-stored water bamboo by retarding weight loss, preserving hardness and TSS, and delaying tissue browning. Integrated transcriptomic and metabolomic analyses demonstrated that this preservation effect was primarily achieved through the coordinated downregulation of the phenylpropanoid and lignin biosynthetic pathways. EBR treatment delayed lignification in water bamboo during refrigeration by suppressing genes associated with lignin biosynthesis, specifically POD, CCR, β-BGL, and *UGT92A1*, as well as related metabolites including L-phenylalanine, (E)-ferulic acid, and shikimic acid. This study not only elucidates the regulatory mechanism of EBR in plant lignification but also provides an applicable postharvest strategy for extending the shelf life and preserving the quality of fresh water bamboo.

## Figures and Tables

**Figure 1:**
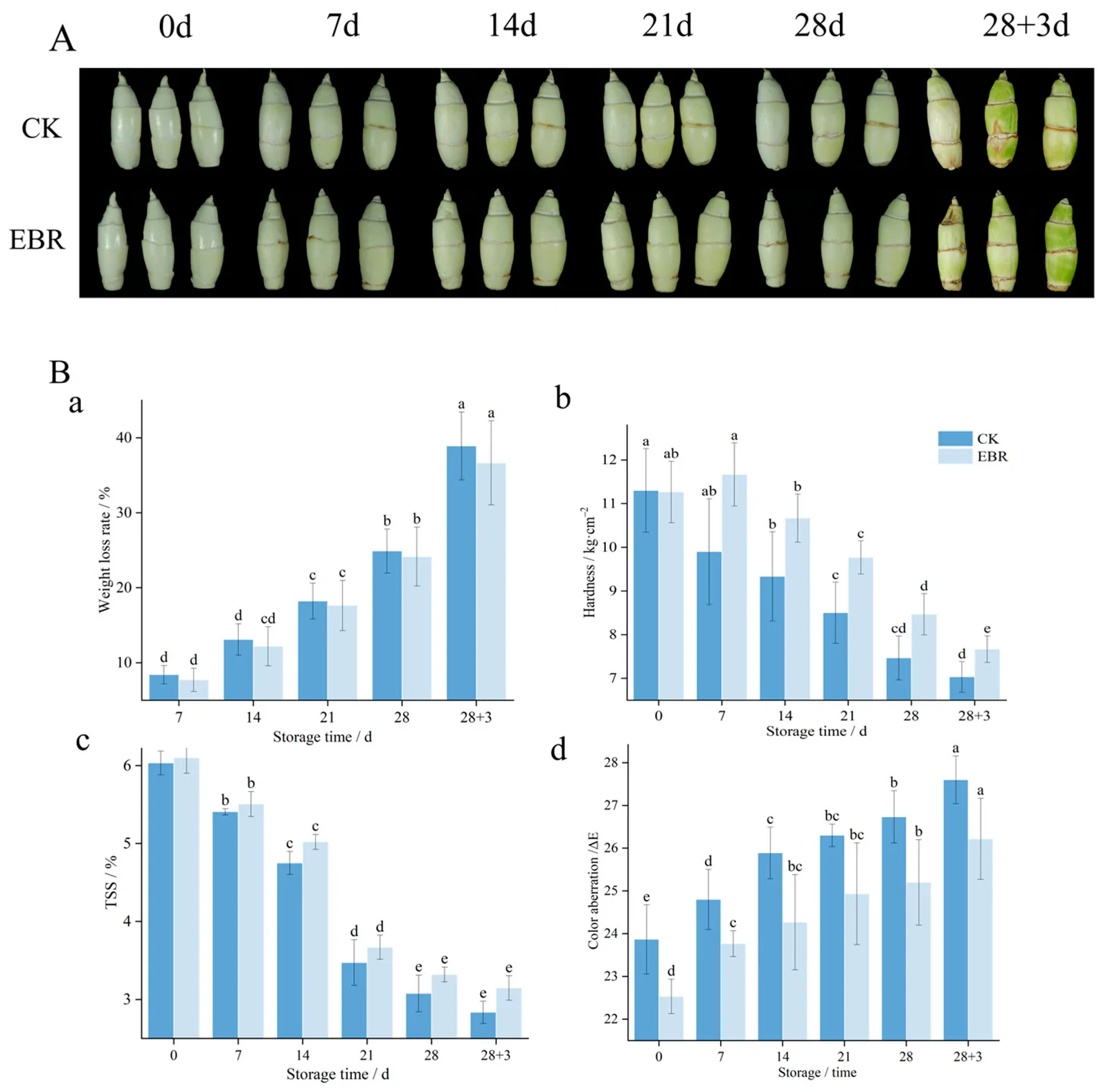
Phenotypic changes in water bamboo before and after cold storage (**A**) and statistical data of quality indicators (**B**). Effects of EBR treatment on weight loss rate (**a**), hardness (**b**), TSS (**c**), and color aberration (**d**) of water bamboo shoots during storage at 4 °C for 28 d and subsequent shelf life at 25 °C for 3 d. Data are presented as mean ± SD (n = 3). Different lowercase letters (a–e) above the bars indicate significant differences among treatments and storage times (*p* < 0.05).

**Figure 2:**
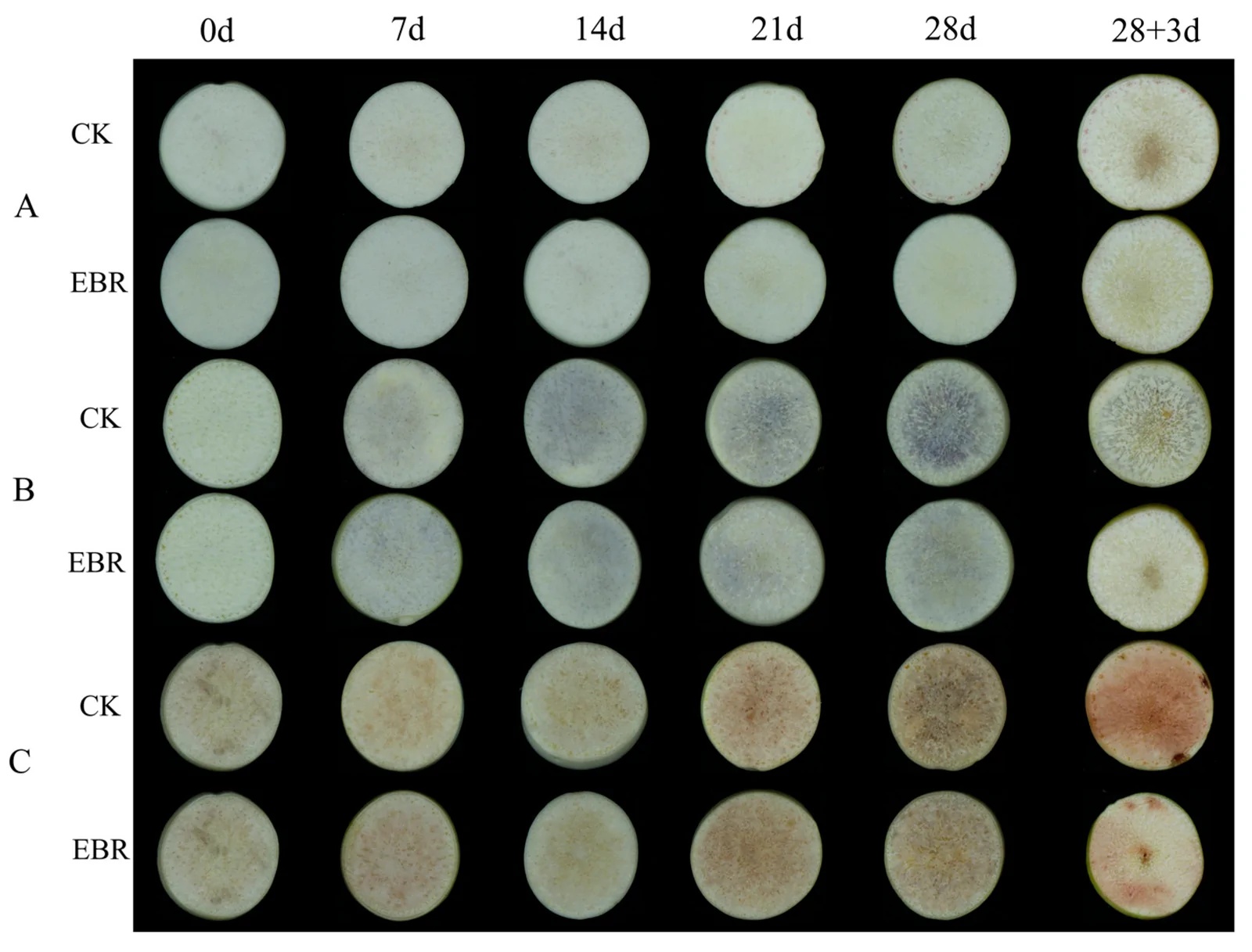
Apparent staining results. Lignin (**A**), O_2_^−^ (**B**), and H_2_O_2_ (**C**) staining of cross-sectional sections.

**Figure 3:**
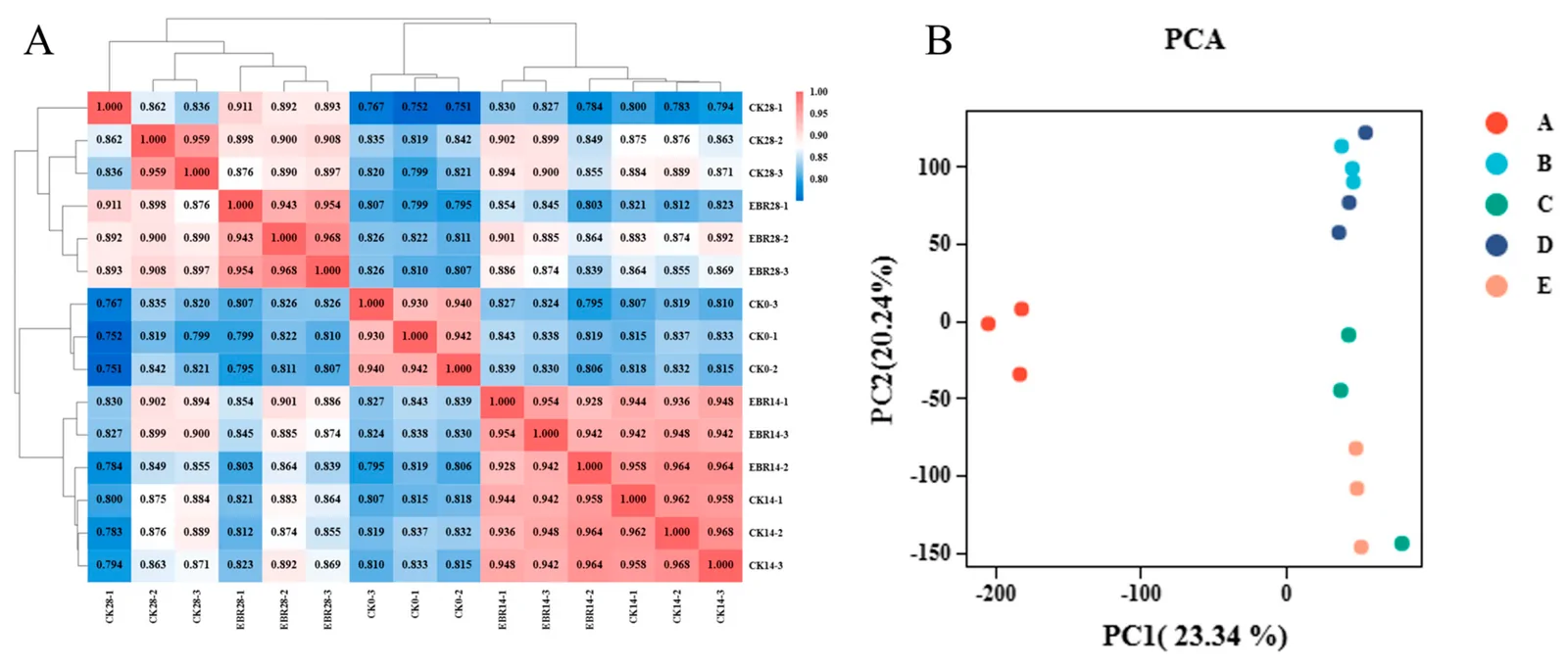
Heatmap of Pearson correlation analysis for gene expression (**A**) and PCA plot (**B**). Groups are designated as follows: CK0 (Group A), CK14 (Group B), CK28 (Group C), EBR14 (Group D), and EBR28 (Group E).

**Figure 4:**
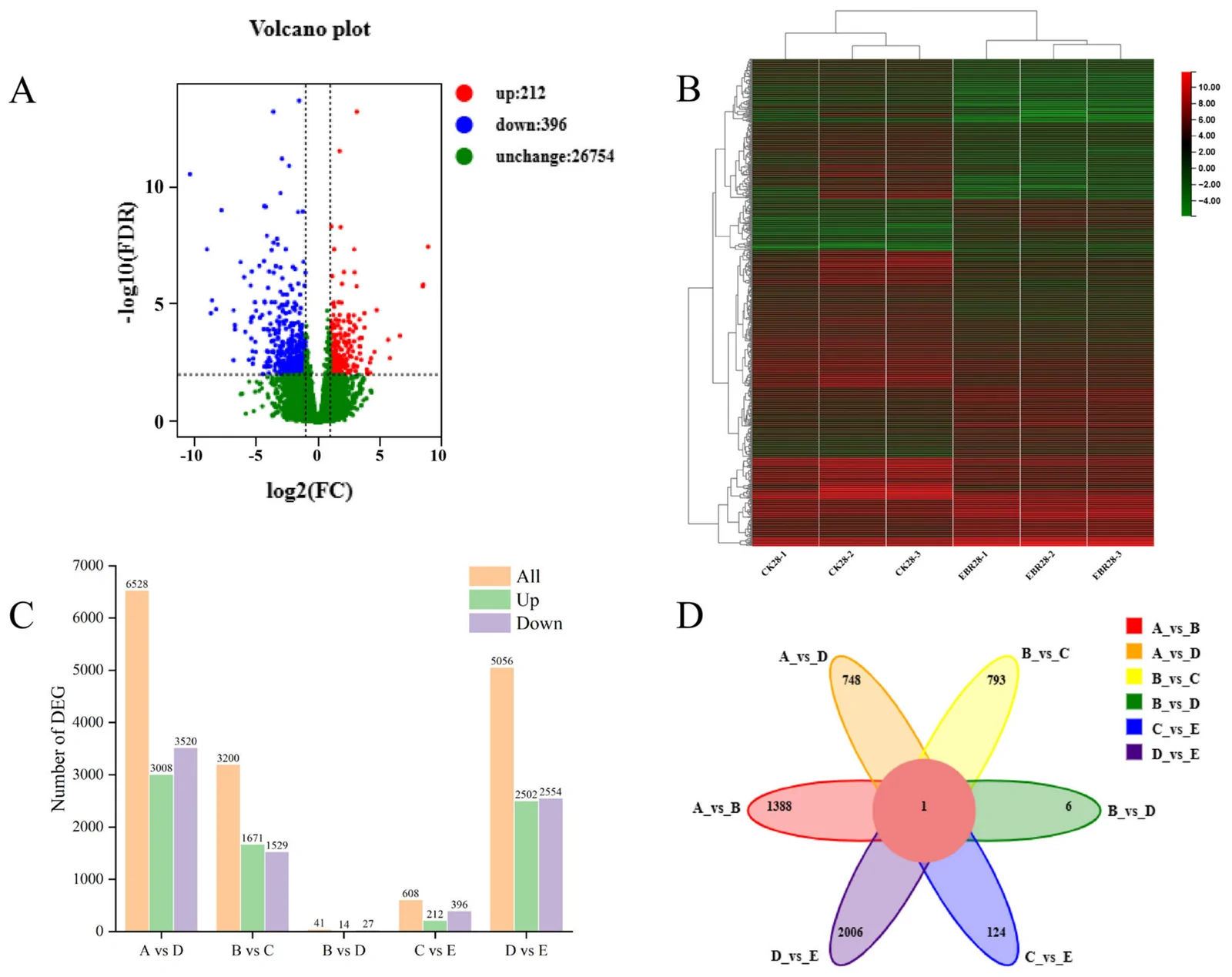
Volcano plot illustrating DEGs between the CK28 and EBR28 groups (**A**), where upregulated and downregulated genes are indicated by red and blue points, and non-differentially expressed genes are shown in green. Hierarchical clustering heatmap of gene expression in the CK28 versus EBR28 comparison (**B**). Up- and downregulation patterns across five groups of DEGs (**C**). A Venn diagram illustrated both the shared and the group-specific DEGs across the five comparison sets (**D**).

**Figure 5:**
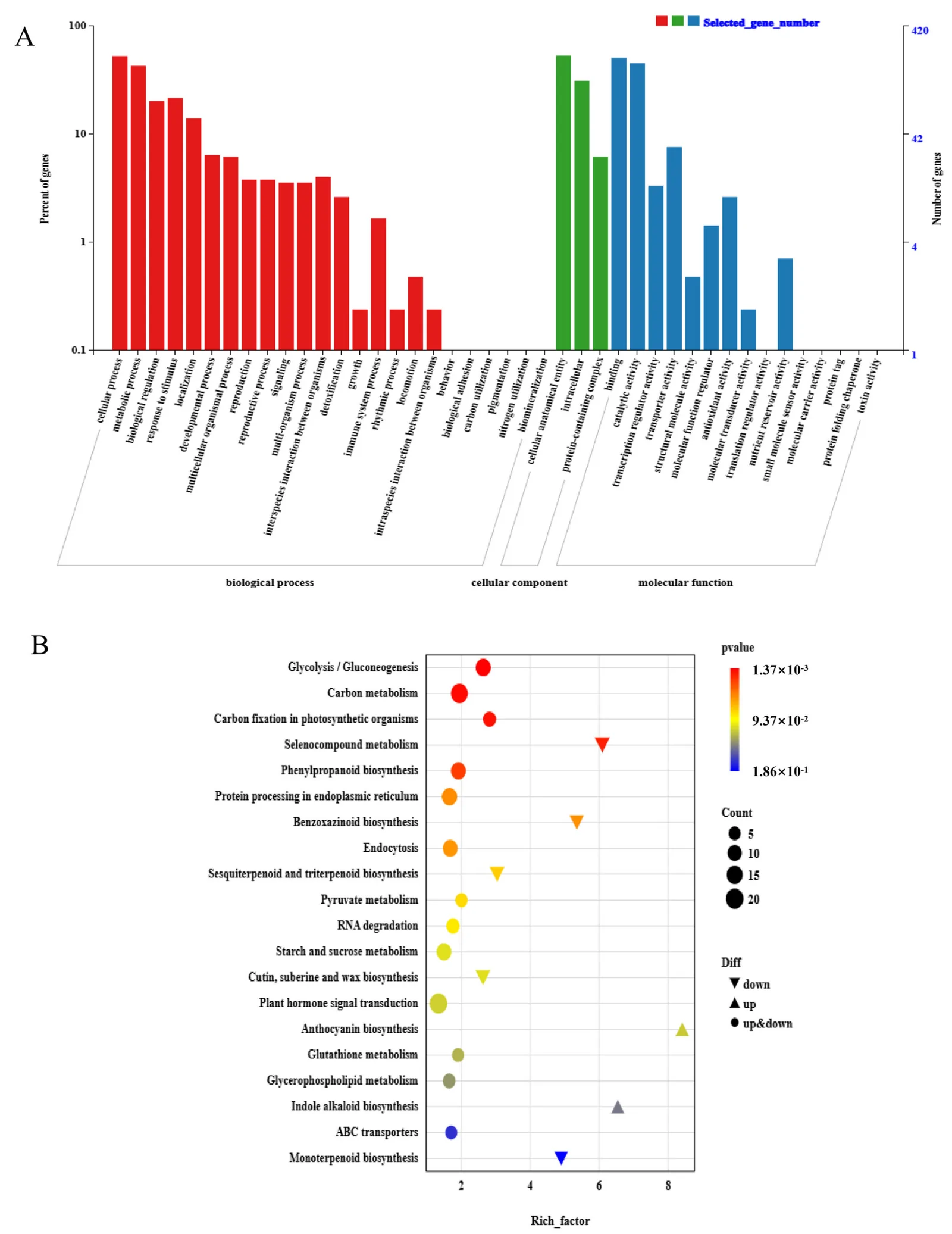
GO classification of DEGs between the CK28 and EBR28 groups (**A**) alongside the KEGG enrichment bubble plot for DEGs (**B**), with significance defined as *p* < 0.05.

**Figure 6:**
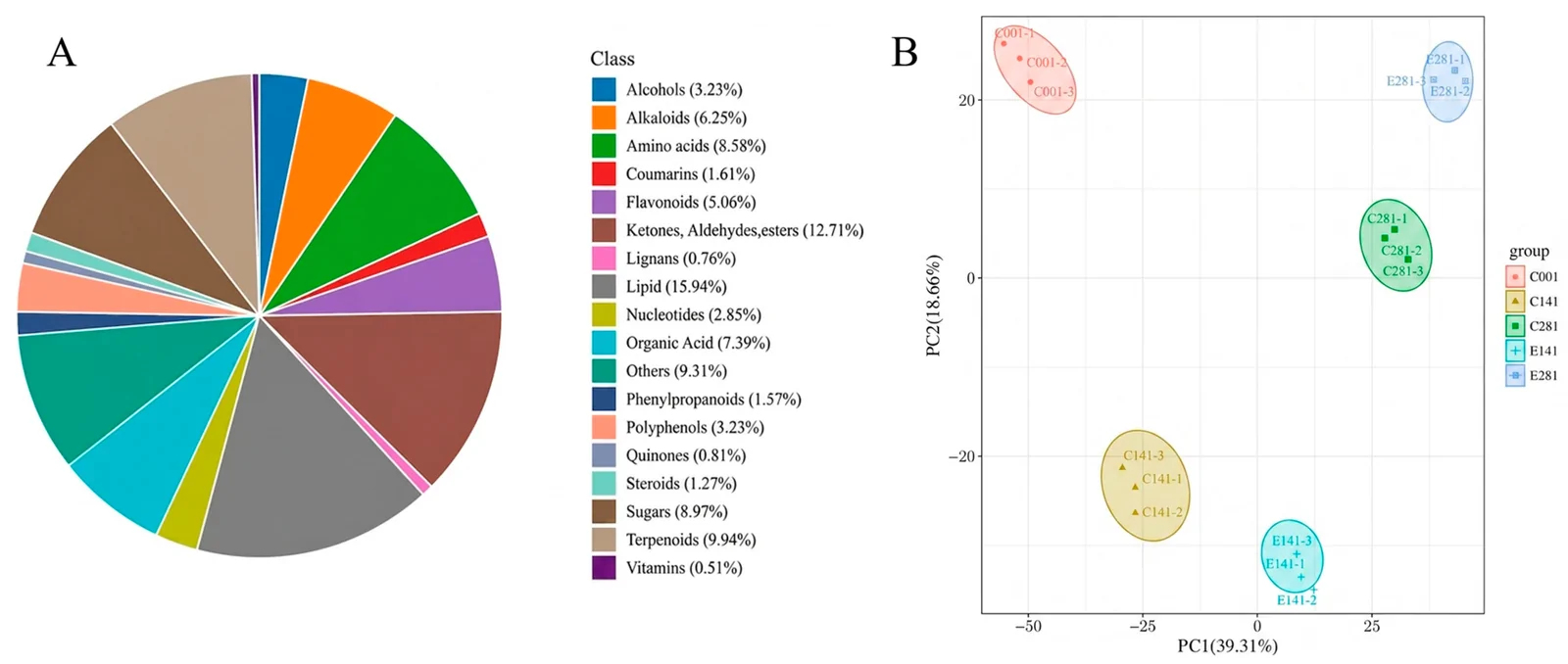
Pie chart of metabolite class composition (**A**), PCA plot of metabolites (**B**).

**Figure 7:**
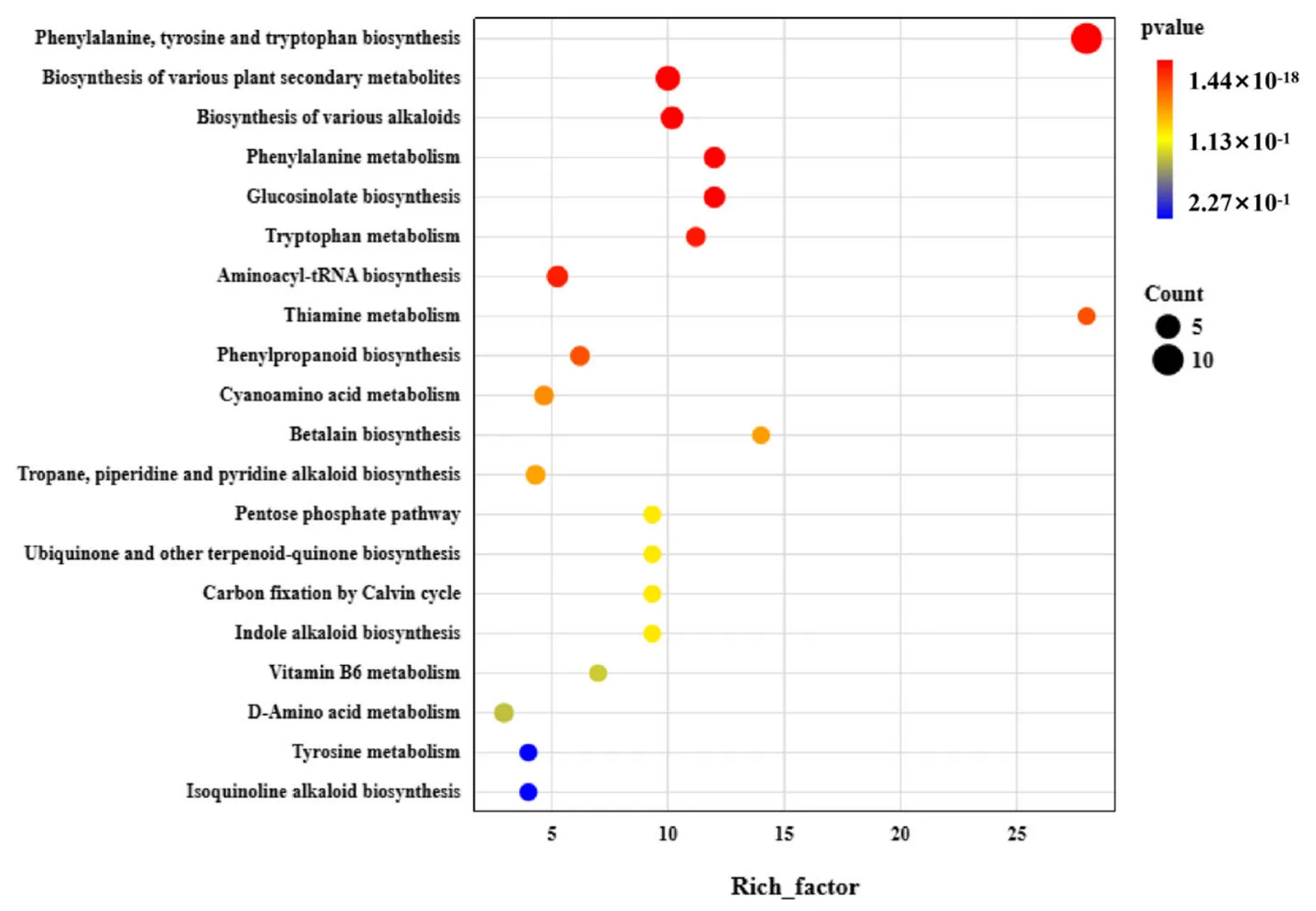
KEGG analysis of DEMs.

**Figure 8:**
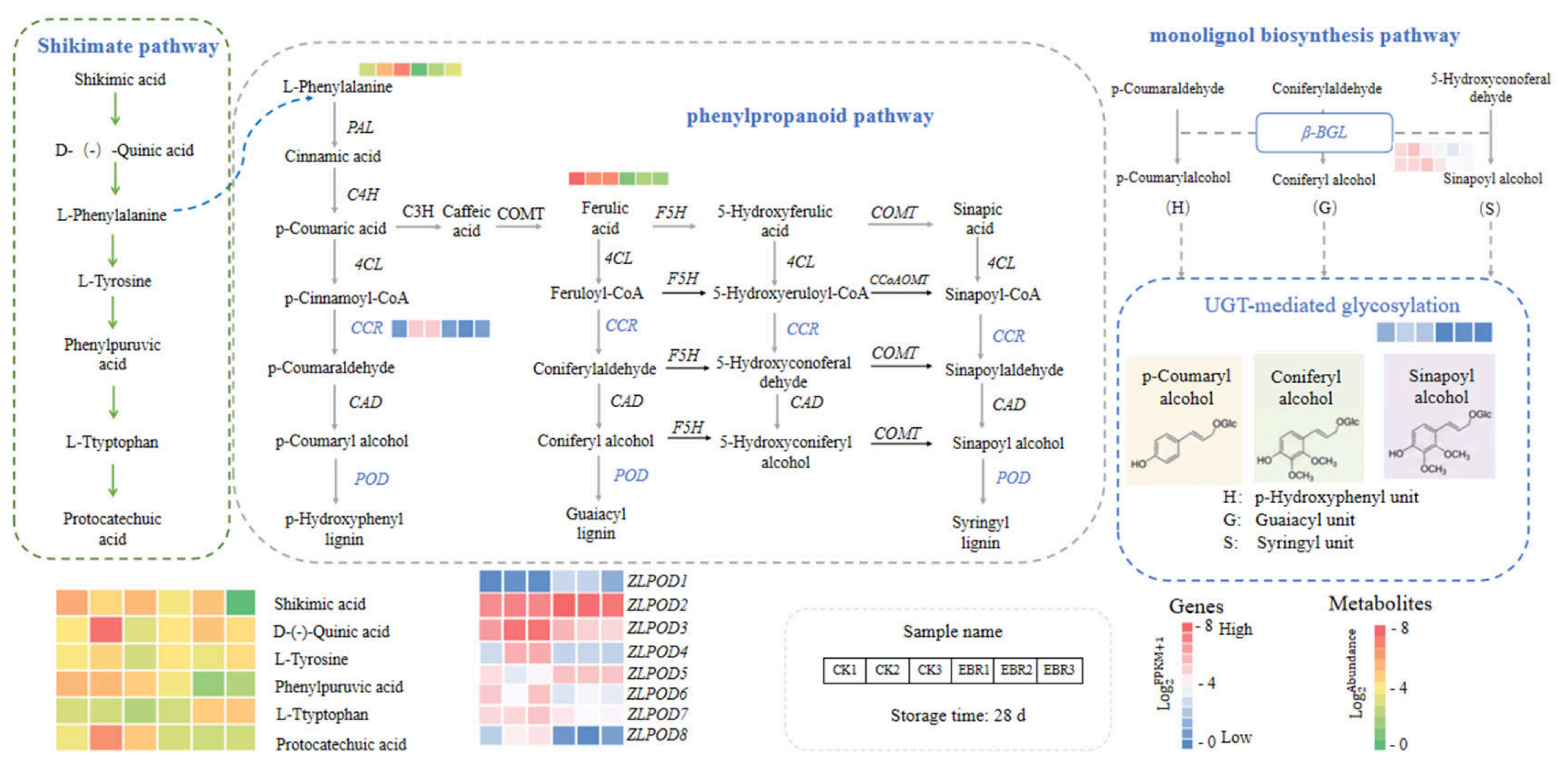
Heatmap of lignin metabolites and gene expression in water bamboo under EBR at day 28 of storage. Metabolites and genes from different omics quantification.

**Table 1:** DEGs related to the phenylpropanoid pathway in water bamboo at day 28 of storage.

ID	Gene Name	Description	log2 FC	Regulated
Zlat_10001910	*POD1*	Peroxidase	3.99	Up
Zlat_10006581	*β-BGL*	β-Glucosidase	−1.94	Down
Zlat_10006654	*POD2*	Peroxidase	1.01	Up
Zlat_10008844	*POD3*	Cationic peroxidase SPC4	−3.04	Down
Zlat_10009342	*POD4*	Peroxidase	−3.96	Down
Zlat_10015519	*POD5*	Peroxidase	1.79	Up
Zlat_10020177	*POD6*	Peroxidase	−2.16	Down
Zlat_10025030	*POD7*	Peroxidase	−2.61	Down
Zlat_10032799	*β-BGL*	β-Glucosidase	−1.42	Down
Zlat_10036302	*POD8*	Peroxidase	−4.74	Down
Zlat_10038007	*CCR*	Cinnamoyl-CoA Reductase	−5.42	Down
Zlat_10047075	*UGT92A1*	UDP-Glycosyltransferase 92A1	−3.91	Down

## Data Availability

The original contributions presented in this study are included in the article. Further inquiries can be directed to the corresponding authors.
